# Influence of Copigmentation and Encapsulation on Stability and Antioxidant Activity of Anthocyanins from Blue and Pink Cornflower (*Centaurea cyanus* L.) Flowers

**DOI:** 10.3390/molecules30071467

**Published:** 2025-03-26

**Authors:** Aleksandra Popowska, Joanna Oracz

**Affiliations:** Institute of Food Technology and Analysis, Faculty of Biotechnology and Food Sciences, Lodz University of Technology, 2/22 Bohdana Stefanowskiego Street, 90-537 Lodz, Poland; popowska.aleksandra@o2.pl

**Keywords:** copigmentation, encapsulation, anthocyanins, cornflower

## Abstract

The aim of this study was to determine the optimal conditions for copigmentation and encapsulation of anthocyanins extracted from blue and pink cornflower (*Centaurea cyanus* L.) flowers and to produce encapsulates resistant to environmental factors. Extracts rich in anthocyanins were encapsulated using spray-drying and sublimation techniques. Baicalin and chlorogenic acid were successfully used as copigmentation agents for blue and pink cornflower anthocyanins. The extracts and encapsulates obtained were subjected to stability tests to assess the stability of color and changes in total anthocyanins content, total phenolic compounds content, and antioxidant activity under exposure to high temperature and UV radiation. The developed encapsulation method effectively protects anthocyanins from adverse environmental conditions. The obtained preparations were characterized by a high content of anthocyanins and phenolic compounds as well as a strong antioxidant potential. The highest stability was demonstrated by anthocyanin encapsulates isolated from the pink flowers of the cornflower, obtained by spray-drying with the addition of a copigment (chlorogenic acid or baicalin) and with a weight ratio of anthocyanin extract to carrier of 1:10. The results of this study suggest that anthocyanin preparations from cornflower, after encapsulation and copigmentation with baicalin or chlorogenic acid, can be used as stable colorants in the food industry as well as functional ingredients providing high levels of antioxidant activity.

## 1. Introduction

Anthocyanins, one of the most important bioactive compounds, are found in flowers, fruits, and tubers. They are natural pigments with bioactive properties and are widely used in the food industry. The most common natural source of anthocyanins are red/blue vegetables and fruits, although levels vary from species to species depending on variety, growing climate, seasonal variation, method of plant cultivation, ripening, processing and storage, temperature, and light intensity [1,2,3]. Edible flowers such as hibiscus, red clover, rose, and cornflower are also rich sources of anthocyanins [2].

Edible flowers are gaining importance due to the growing interest in natural products rich in bioactive health-promoting compounds such as anthocyanins. Anthocyanins belong to the group of water-soluble flavonoids. Structurally, these compounds are glycosidic derivatives of the corresponding anthocyanidins [1,2,3]. They are used not only in the food industry as natural pigments, but also in the production of medicinal and pharmaceutical products, such as ointments, tinctures, infusions, or herbal mixtures [1,2,3].

Their attractive colors make them of great interest, but their low stability limits their widespread use. The stability of anthocyanins depends on factors such as pH, chemical structure, and the presence of metal ions or copigments. Degradation of these compounds can occur in several ways, including chemical reactions, cleavage, or polymerization, which changes the color and properties of the compound [3].

In the food industry, artificial colors are increasingly being replaced by natural pigments such as chlorophylls, carotenoids, and anthocyanins. Natural pigments are more valued because they have health-promoting properties and a lower risk of causing adverse reactions such as allergies or hyperactivity in children. Extraction of anthocyanins requires appropriate extraction methods to minimize their degradation. The efficiency of the process is influenced by the temperature, the type of solvent, and the material/solvent ratio, among other factors. Modern techniques, such as ultrasound-assisted extraction, help to reduce the time, energy, and amount of solvent used [4].

To increase the stability of anthocyanins, new methods are being developed to protect these compounds. One such method is encapsulation, in which the active compound is enclosed in protective coatings. In this process, small molecules are surrounded by a carrier material that provides stability and controlled release of the substance. Popular encapsulation methods include spray-drying and lyophilization. These methods are widely used in the production of microcapsules, powdered emulsions, and hydrogels. Spray-drying is fast and efficient, and carriers such as maltodextrin, starch, gelatin, or arabic gum are used to form coatings. However, it has been shown that the use of heat during spray-drying significantly reduces anthocyanin retention, especially for molecules located on the outer surface of the microcapsules [5,6]. Lyophilization is ideal for preserving flavors and heat-sensitive substances, although it is more expensive and time-consuming. Nevertheless, freeze-dried encapsulated products have greater anthocyanin retention compared to those produced by spray-drying, which is likely due to the lower temperatures involved in the freeze-drying process [7]. Both methods are widely used in the food industry to encapsulate vitamins, colors, minerals, or flavors [5,6,7].

Storage and processing conditions have a significant impact on the stability of anthocyanins. One method of stabilizing anthocyanins and increasing their color intensity is co-pigmentation. Copigmentation is a phenomenon that results in the formation of pigments or molecular complexes. It is due to the molecular bonds between pigments and other organic molecules, which are usually colorless [8]. It is based on the interaction of intermolecular, electrostatic hydrogen bonds and hydrophobic interactions. It refers to a phenomenon that significantly affects the color intensity and stability of anthocyanins by combining them with flat unsaturated moieties. A number of colorless compounds such as hydroxybenzoic and hydroxycinnamic acids and hydroxyflavones can act as copigments. These compounds are naturally colorless, but they intensify the color of anthocyanin solutions. Therefore, temperature, pH, structure, and the presence of other molecules (copigments) play an important role in the coloration of anthocyanins in solution. Copigmentation is widely used in the food industry to adjust the color of foods. The goal is to maintain or reproduce the natural color intensity or to create a new shade [1,8,9]. It is the most common stabilization mechanism. Copigmentation of anthocyanins with phenolic acids and flavonoids, such as baicalein and its derivatives, has been shown to increase the color intensity of flowers [2].

Despite the fact that cornflowers (*Centaurea cyanus* L.) can be an excellent source of anthocyanins, so far there have been only a few studies [10,11,12] on the process of obtaining stable preparations of anthocyanins from this raw material by encapsulation. Pink and blue cornflower flowers are an excellent source of several anthocyanin types, mainly in the form of acetyl glycosides attached to aglycones, such as cyanidin and pelargonidin [10]. These compounds are usually present in small amounts in berries and fruits, which are the main source of natural pigments. Acetylation increases the intensity and stability of anthocyanins, making them more suitable for use as natural colorants and bioactive components in functional foods, cosmetics, and pharmaceuticals [13]. Therefore, the aim of this work was the encapsulation of anthocyanins from blue and pink cornflower flower extracts by both spray-drying and freeze drying using a mixture of maltodextrin and acacia fiber as carriers and baicalin and chlorogenic acid as copigments. The composition of bioactive compounds, including anthocyanins and phenolic compounds by UHPLC-DAD-ESI-MS/MS and spectroscopic methods, antioxidant capacities using DPPH and FRAP tests, and stability under thermal and light treatments of the obtained encapsulates, were evaluated to provide valuable information and identify the most suitable encapsulation and copigmentation technique in terms of potential applications in the food, cosmetic, or pharmaceutical industries.

## 2. Results and Discussion

### 2.1. Characterization of Anthocyanin Extracts from Blue and Pink Cornflower Flowers

In the first part of the study, the qualitative and quantitative composition of anthocyanins and main phenolic compounds in two varieties of cornflower (*C. cyanus*) was characterized using the UHPLC-DAD-ESI-MS/MS method (Table 1). The blue and pink cornflower flowers were characterized by a different anthocyanin profile. In the extract of blue cornflower petals, cyanidin 3,5-diglucoside (Cy3G5G) and its derivatives acetylated with malonic acid (cyanidin 3-*O*-(6″-malonyl-glucoside)-5-*O*-glucoside, Cy3malonylG5G) and succinic acid (cyanidin 3-*O*-(6″-*O*-succinyl-glucoside)-5-*O*-glucoside, Cy3succinylG5G) were determined. In the extract of pink cornflower flowers, mainly pelargonic acid derivatives were identified, including pelargonidin 3,5-di-*O*-glucoside (Pg3G5G), pelargonidin 3-*O*-(6″-malonyl-glucoside)-5-*O*-glucoside (Pg3malonylG5G), pelargonidin 3-*O*-(6″-*O*-succinyl-glucoside)-5-*O*-glucoside (Pg3succinylG5G), pelargonidin 3-*O*-malonyl-glucoside-5-*O*-malonyl-glucoside (Pg3malonylG5malonylG), and pelargonidin 3-*O*-malonyl-glucoside-5-*O*-succinyl-glucoside (Pg3malonylG5succinylG). As with the blue cornflower, the extract from the pink variety also contained Cy3malonylG5G. Other phenolic compounds that were identified in the extracts of the blue and pink cornflower flowers were chlorogenic acid (ChA) and apigenin 7-*O*-glucuronide (Ap-GluA). The results obtained are consistent with data presented by other authors [10].

**Figure 1:**
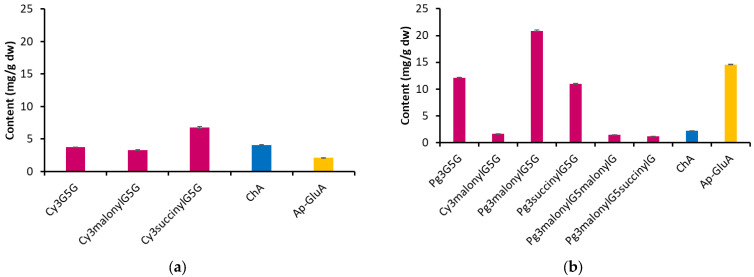
The content of individual anthocyanins and other phenolic compounds in the extracts of blue and pink cornflower flowers (mg/g dry weight), (**a**) blue variety, (**b**) pink variety.

Figure 1 shows the results of determining the concentration of individual anthocyanins and other phenolic compounds identified in extracts from the flowers of blue and pink cornflowers. It was observed that the extract from the flowers of the pink variety of cornflower contains significantly more anthocyanins than extract from the flowers of the blue variety. In extract from blue cornflower flowers, the dominant compound was Cy3succinylG5G (6.79 mg/g dw), followed by Cy3G5G (3.72 mg/g dw) and Cy3malonylG5G (3.28 mg/g dw). In the extract of the pink variety, Pg3malonylG5G (20.87 mg/g dw) was present in the highest concentration. It was followed by Pg3G5G (12.10 mg/g dw) and Pg3succinylG5G (11.94 mg/g dw). The other anthocyanins identified were present at much lower levels (<2 mg/g dw). The content of the other two identified phenolic compounds differed between the extracts obtained from different cornflower flowers.

Chlorogenic acid was present in higher amounts (4.09 mg/g dw) in the blue cornflower extract, while apigenin 7-*O*-glucuronide was present in much higher concentrations in the pink cornflower extract (14.09 mg/g dw).

Table 2 shows the total anthocyanins, total phenolic compounds content, and antioxidant activity of extracts from different cornflower cultivars. The total content of the tested compounds and the antioxidant activity of the extracts obtained varied significantly (*p* < 0.05).

The anthocyanins and total phenolic compounds concentration in the extract from the flowers of the pink variety was almost four times higher than in the extract from the blue flowers. The capacity for scavenging free radicals and the ability to reduce ferric ions were also greater in the pink cornflower extract (511.88 and 485.81 μM TE/g dw, respectively) than in the blue cornflower extract (402.19 and 383.47 μM TE/g dw, respectively). In this study, the antioxidant activity of extracts obtained from blue and pink varieties of cornflower was determined for the first time. However, in comparison with the results of other studies [11,12], the antioxidant activity of the obtained extracts is more than two to almost 10 times higher for the extract from blue and pink cornflower flowers, respectively.

### 2.2. Copigmentation and Encapsulation of Anthocyanins from Blue and Pink Cornflowers

**Figure 2:**
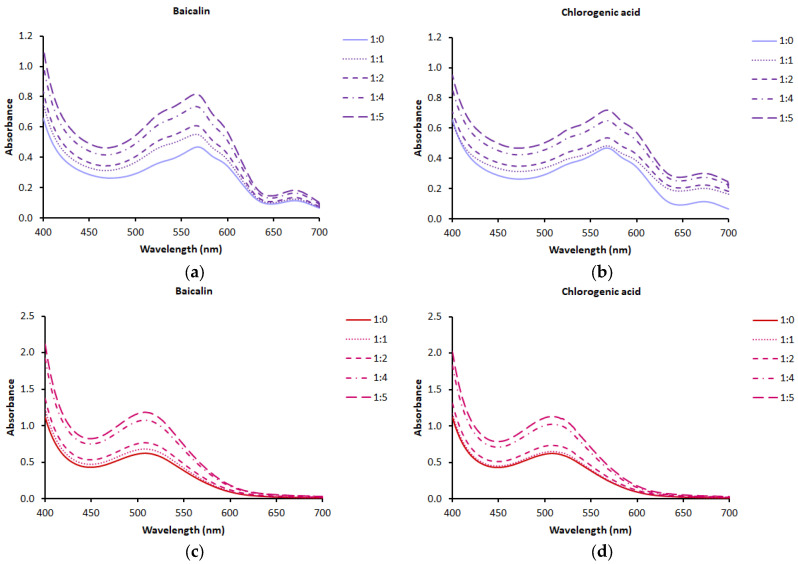
Absorption spectra of anthocyanins/copigments solutions in different molar ratios: (**a**) anthocyanins from blue cornflower/baicalin solutions; (**b**) anthocyanins from blue cornflower/chlorogenic acid solutions; (**c**) anthocyanins from pink cornflower/baicalin solutions; (**d**) anthocyanins from pink cornflower/chlorogenic acid solutions.

One aspect of the research was to determine the conditions for the copigmentation reaction of anthocyanins from blue and pink cornflower extracts. Based on literature data, baicalin and chlorogenic acid were selected as copigments [1,14,15,16,17]. The copigmentation reaction of anthocyanins in the form of cornflower extracts was carried out at room temperature for 30 min in solutions at pH 3.5. It has been shown that the anthocyanins present in cornflower extracts are susceptible to copigmentation reactions and can undergo structural transformations under the conditions used. After the addition of the copigment to the anthocyanin solution, the spectral properties of the solution were modified, affecting the absorbance intensity at the wavelengths characteristic of the anthocyanins present in the extracts studied (Figure 2). The change in color intensity was probably the result of a shift in the hydration equilibrium towards the flavin form, due to the selective interaction of anthocyanins with the copigment [1].

The highest increase in absorbance intensity at the wavelength characteristic of anthocyanins present in blue and pink cornflower extract was observed when baicalin was used as a copigment. A slightly lower increase in absorbance intensity was found when chlorogenic acid was used as a copigment. Regardless of the copigment used, the highest increase in absorbance intensity was found when the molar ratio of cornflower blue and pink anthocyanins to copigment was 1:5.

The next stage of the research was to develop a method for stabilizing anthocyanins from blue and pink cornflower flowers. Based on a review of the literature [14,15,16,17,18], the method of spray-drying and freeze-drying was used to stabilize anthocyanins in the form of extracts obtained from the tested flowers. A mixture of low-sugar maltodextrin (DE 7-13%) and accai fiber (in a 1:1 weight ratio) was used as the coating agent. These polysaccharides were used as carrier agents due to their chemical nature and encapsulating properties, including emulsifying capacity, low viscosity, water solubility, and low hygroscopicity [19]. The encapsulates were obtained by spray-drying and freeze-drying in which the weight ratio of extract to carrier was 1:4 or 1:10. In the case of encapsulation and copigmentation, the molar ratio of anthocyanins to copigment was 1:5.

Table 3 shows the results of the determination of total anthocyanins, total phenolic compounds, and antioxidant potential of encapsulated anthocyanin extracts of blue and pink cornflower flowers.

As expected, the encapsulates have a lower content of anthocyanins and phenolic compounds than the extracts. The content of these compounds in the encapsulates obtained by both spray-drying and freeze-drying depended on the weight ratio of extract to carriers and the type of copigment used. The use of copigments did not have a significant effect on the anthocyanin concentration in the obtained preparations, but it had a significant effect on the total phenolic compounds content. It was also found that the encapsulates obtained by spray-drying had a slightly lower content of anthocyanins and phenolic compounds compared to their concentration in encapsulates obtained by a freeze-drying method. In freeze-drying, frozen preparations are dehydrated by sublimation so that the dried substances are not exposed to high temperatures, thus better preserving thermolabile compounds such as anthocyanins. Spray-drying, on the other hand, removes water quickly, so this technique can also be used to encapsulate anthocyanins, although the loss of these pigments is slightly higher. In addition, the time and cost of the spray-drying method are significantly lower than those of freeze-drying [5,6,7]. Therefore, the spray-drying method would be more advantageous than freeze-drying for the industrial application of the developed encapsulation and copigmentation method.

It has also been shown that combining the encapsulation and copigmentation methods increases the antioxidant activity of the obtained anthocyanin preparations from the flowers of blue and pink cornflowers. The addition of copigments, such as baicalein and chlorogenic acid, significantly increased the free radical scavenging capacity and reducing power of encapsulates from cornflowers. The lowest ability to scavenge the free stable DPPH radical (122.66 μM TE/g dw) and the lowest ferric ion-reducing/antioxidant power (111.81 μM TE/g dw) had anthocyanin encapsulation from blue cornflower, which was obtained by spray-drying with a 1:10 extract-to-carrier ratio. The reduction in the antioxidant capacity is probably due to the high encapsulation efficiency, which leads to the blocking of active groups of compounds with antioxidant properties. A reduction in the weight ratio of the carrier to the extract and the use of a copigment contributed to a significant increase in the antioxidant potential, even in relation to the initial extract. The highest free radical scavenging capacity (666.58 μM TE/g dw) and ferric reducing ability (491.33 μM TE/g dw) was exhibited by pink cornflower anthocyanin encapsulates obtained by freeze-drying using a 1:4 extract/carrier weight ratio and baicalin as copigment.

The preparations obtained according to the developed method of encapsulation and copigmentation of anthocyanins from two different varieties of cornflower were characterized by higher content of phenolic compounds and higher antioxidant potential in comparison with the data presented by other authors [11,12]. Nevertheless, these studies also show that the total phenolic content and antioxidant activity of cornflower petal encapsulates depend on the type and ratio of carriers. However, these preparations were obtained only by freeze-drying and using only one variety of cornflower.

In this study, for the first time, extracts from different varieties of cornflower have been encapsulated and copigmented using a spray-drying method.

### 2.3. Stability of Cornflower Anthocyanin Extracts and Encapsulates

Table 4 shows the results of determination of the color stability index after incubation of the extracts and encapsulates at 60 °C for 72 h (thermal treatment) and affected by UV-A radiation (light treatment). The changes in anthocyanin color that occur during thermal and light treatments of the tested preparations were assessed by determining the color stability index.

Anthocyanins in the form of non-encapsulated extracts were characterized by the lowest thermal and light stability. The use of encapsulation and copigmentation contributed to an increase in anthocyanin color stability, as evidenced by the increase in the stability index from 0.61–0.70 observed for the original extracts to 0.82–0.94 for the encapsulates. Light treatment of extracts and encapsulates obtained from blue and pink cornflower flowers had a significant effect on their color. Although the anthocyanin stability observed upon exposure to light showed similar behavior, the color change was greater for the extract and encapsulates obtained from blue cornflowers. In addition, it was observed that the light color stability was better compared to thermal treatments.

The highest thermal stability was shown by the encapsulates of anthocyanins isolated from pink cornflower flowers, obtained by spray-drying with the addition of a copigment (chlorogenic acid or baicalin) and with a weight ratio of anthocyanin extract to carrier of 1:10. The results obtained are in agreement with the studies of other authors [20], who also indicated that phenolic compounds used as copigments allow to increase the maximum absorption of anthocyanins, as well as to reduce the rate of thermal degradation. The difference in the color stability index was more pronounced at high temperatures than under the influence of UV radiation. In the case of free anthocyanins, this phenomenon causes a loss of color and the appearance of brown compounds due to the formation of chalcone [21,22]. On the other hand, the use of encapsulation with a mixture of maltodextrin and acacia fiber significantly reduces the problem of color change and protects unstable anthocyanins from degradation at high temperatures.

**Figure 3:**
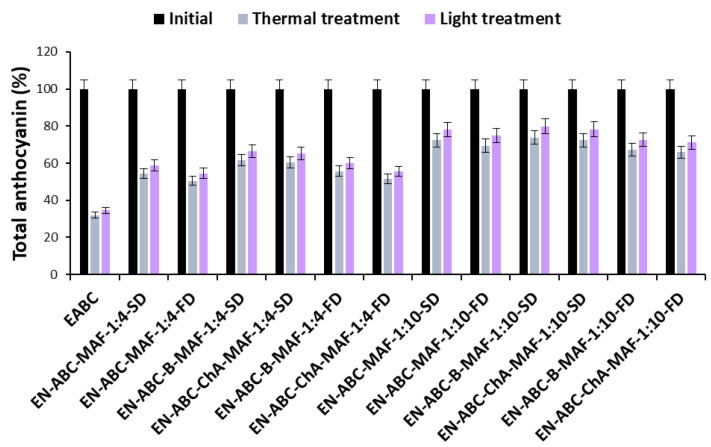
Changes in the total anthocyanin content of blue cornflower extracts and encapsulates during thermal and light treatments, expressed as percentage.

**Figure 4:**
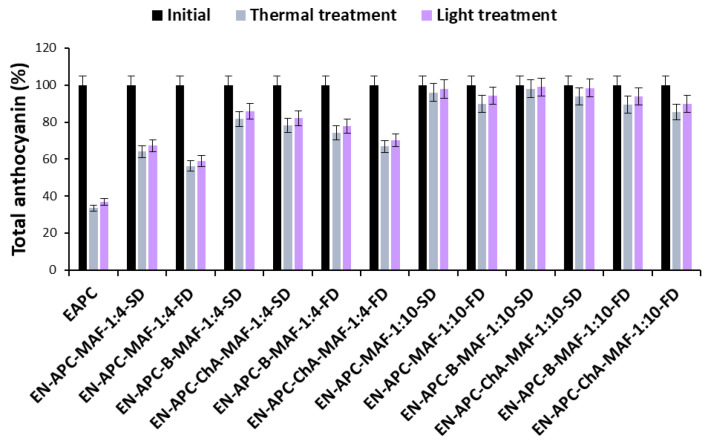
Changes in the total anthocyanin content of pink cornflower extracts and encapsulates during thermal and light treatments, expressed as percentage.

**Figure 5:**
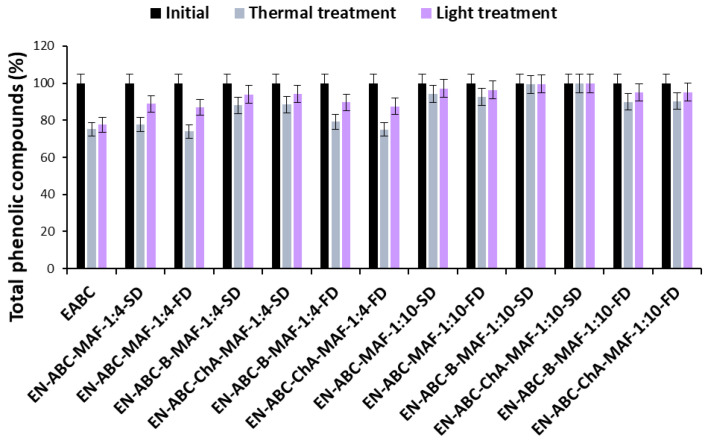
Changes in the total phenolic compounds content of blue cornflower extracts and encapsulates during thermal and light treatments, expressed as percentage.

**Figure 6:**
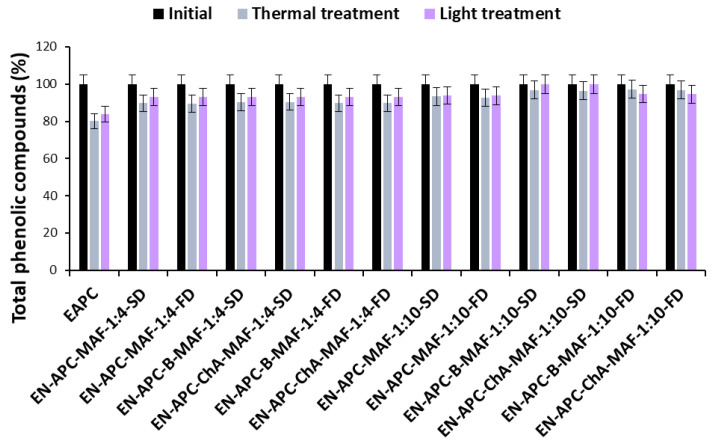
Changes in the total phenolic compounds content of pink cornflower extracts and encapsulates during thermal and light treatments, expressed as percentage.

Changes in the total anthocyanin and phenolic content of blue and pink cornflower anthocyanin extracts and encapsulates are shown in Figure 3, Figure 4, Figure 5 and Figure 6.

A decrease in anthocyanin concentration was observed in cornflower extracts and encapsulates after heat and light treatment. A slightly greater decrease in anthocyanin content was observed under high temperature than under UV light. These changes may be due to partial degradation and structural transformation of anthocyanins [22,23,24]. In addition, the anthocyanins from pink cornflower were found to be more stable than the compounds present in the blue cornflower extract. These results may be related to the diverse stability of different anthocyanin groups [22]. In the pink cornflower, the predominant group of anthocyanins was the pelargonidin derivatives, which are more stable than the cyanidin derivatives—the main anthocyanins of the blue cornflower. This phenomenon may be caused by the fact that hydroxylation of the B-ring in the anthocyanin structure reduces its stability. Therefore, cyanidin derivatives with two hydroxyl groups on the B-ring are less stable than pelargonidin derivatives with only one hydroxyl group on the B-ring [3].

Based on the results, it was shown that the use of encapsulation and copigmentation processes reduces the loss of total phenolic compounds in the preparations. The highest loss of total phenolic compounds was observed in extracts and in encapsulates where the weight ratio of extract to excipient was 1:4. The lowest loss of total phenolic compounds was observed in encapsulates with the highest carrier content. These preparations are more stable, indicating good copigmentation efficiency to reduce losses after thermal and light treatments. It was also found that the changes in the levels of phenolic compounds were much smaller than in the case of anthocyanins.

These results are in line with studies by other authors [25,26] which have also shown that the stability of anthocyanins is significantly increased after encapsulation.

## 3. Materials and Methods

### 3.1. Materials

Gallic acid, baicalin, chlorogenic acid, cyanidin 3-O-glucoside chloride, acetonitrile of HPLC grade (≥99.9%), 6-hydroxy-2,5,7,8-tetramethylchroman-2-carboxylic acid (Trolox), 2,2-diphenyl-1-picrylhydrazyl (DPPH), 2,4,6-tri(2-pyridyl)-s-triazine (TPTZ), sodium acetate, iron(III) chloride hexahydrate were all obtained from Sigma-Aldrich (St. Louis, MO, USA). Water was purified by a Milli-Q water purification system (Millipore Corp., Bedford, MA, USA). All other chemicals were of analytical grade, and reagents were prepared according to standard analytical procedures.

Plant materials used in the study were dried blue and pink cornflower flowers (Coffee Roasters 1995, Tychy, Poland), maltodextrin N (Pepees S.A., Łomża, Poland), and acacia fiber (Biomus, Lublin, Poland).

### 3.2. Preparation of Anthocyanin Extracts

The extraction of anthocyanins from cornflower flowers was carried out according to the method of Carrera i in. (2021) [27]. Approximately 30 g of crushed flowers were placed in a 250 mL glass bottle, and 450 mL of 20% acidified methanol (pH 2) was added. The mixture was sonicated at up to 40 °C for 30 min. The extraction conditions, solvent composition, and pH used were selected based on preliminary tests, which showed that these were the optimal conditions for the most effective extraction of anthocyanins from cornflower flowers. After extraction, the solution was filtered through a filter paper into a clean 1000 mL glass bottle. Methanol was evaporated with nitrogen, and the aqueous extract was frozen and freeze-dried in a DELTA 1-24LSC freeze dryer (Martin Christ, Osterode am Harz, Germany) at −50 °C, vacuum 0.7 mbar for 48–72 h. The dried extracts were ground and stored at −20 °C in sealed containers until further use.

### 3.3. Copigmentation of Anthocyanin Extracts

The copigmentation process was performed according to Zhang et al. (2016) [17] with some modifications. An anthocyanin solution was prepared by dissolving extract of blue or pink cornflower in 0.1 M HCl solution (pH 3.5) so that the resulting solution had a concentration of 3 mg/mL. Chlorogenic acid and baicalin were used as copigments. The prepared anthocyanins extract solutions were mixed with copigment solutions to obtain anthocyanin/copigment molar ratio of 1:0, 1:1, 1:2, 1:4, and 1:5. Then, the mixtures were placed in an orbital shaker and shaken at room temperature at 80 rpm for 30 min. After completion of the copigmentation reaction, the UV-Vis absorption spectra of the resulting solutions were recorded in the 400–700 nm range using a UV-1800 spectrophotometer (Shimadzu, Tokyo, Japan).

### 3.4. Encapsulation of Anthocyanin Extracts

The encapsulation process of anthocyanins from the flowers of blue and pink cornflower was developed based on the procedure described by Vergara et al. (2016) [10], with some modification.

#### 3.4.1. Preparation of Solutions

First, cornflower anthocyanin extract or cornflower anthocyanin extract with copigment (anthocyanin/copigment molar ratio of 1:5) was mixed with 0.1 M HCl (pH 3.5) and shaken at 100 rpm for 30 min (1.0% *w*/*w*). Maltodextrin and acacia fiber (in a ratio 1:1) was used as the coating material. A coating solution was prepared by dissolving maltodextrin and acacia fiber in distilled water at 50 °C. The solution was stirred for 30 min. Then, anthocyanin solution or anthocyanin/copigment solution was combined with the coating solution in a ratio 1:4 or 1:10 (*w*/*w*) and stirred continuously at room temperature and protected from light for 18 h.

#### 3.4.2. Spray-Drying Processing

The anthocyanin extract solutions with the coating agents mixture was spray-dried using a BÜCHI B-290 laboratory spray-dryer (BÜCHI, Flawil, Switzerland). Drying conditions were selected based on preliminary studies and those performed by other authors [14,15,16,17,18]. Inlet temperature was maintained at 140 °C, while the outlet air temperature was 80 °C. The spray-dryer was equipped with a peristaltic pump operated at 5 mL/min. The dried anthocyanin powder was collected, packed in plastic bags, and stored in airtight containers at −20 °C until analysis.

#### 3.4.3. Freeze-Drying Processing

The anthocyanin extract solutions with the coating agents mixture was frozen and freeze-dried using a DELTA 1-24LSC freeze-dryer (Martin Christ Germany) at −50 °C, vacuum 0.7 mbar for 48–72 h. The dried anthocyanin powder was ground in a mortar and stored in airtight containers at −20 °C until analysis.

### 3.5. Qualitative and Quantitative Analysis of Anthocyanins and Other Phenolic Compounds Using UHPLC-DAD-ESI-MS/MS

Approximately 0.0200 g of dried extracts or encapsulates were weighed into a 15 mL centrifuge tube, and 10 mL of acidified methanol solution (80:20, pH 2) was added. The tubes were sealed, placed on an orbital shaker, and shaken at room temperature at a speed of 80 rpm for 30 min. After this time, the samples were filtered through a syringe filter (with a pore diameter of 0.2 um) into glass vials of the autosampler.

The samples were analyzed by UHPLC-DAD-ESI-MS/MS according to the procedure described by Oracz et al. (2022) [28]. Spectrophotometric detection was performed at 270, 320, 365, and 520 nm. The concentration of individual anthocyanins and phenolic compounds was determined using an external calibration method. The identification of the anthocyanins and other phenolic compounds was based on their retention times, UV-vis spectra, full mass spectra (ESI-MS), and fragmentation spectra (MS/MS), as well as literature data [10,29,30]. The content of each compound was expressed in milligrams per gram of dry weight (mg/g dw).

### 3.6. Determination of Total Anthocyanins According to the Method of Ronald E. Wrolstade

The total anthocyanin content was assessed spectrophotometrically by measuring absorbance at 526 and 700 nm at pH 1 and pH 4.5, following Le et al. (2019) [31]. The total anthocyanin content was expressed in as milligrams of cyanidin 3-*O*-glucoside per gram of dry weight (mg Cy3G/g dw).

### 3.7. Determination of Total Phenolic Compounds by the Folin–Ciocalteu Method

The total phenolic compounds content was determined using the Folin–Ciocalteu method according to the procedure described by Oracz et al. (2023) [32]. The results of the total phenolic content are expressed in milligrams of gallic acid per gram of dry weight (mg GA/g dw).

### 3.8. Thermal Stability of Anthocyanins

The thermal stability of anthocyanins in the form of obtained extracts and encapsulates from the flowers of cornflower of the blue and pink varieties was carried out using the method described by Vergara et al. (2020) [18].

Approximately 0.0400 g of dried extract or encapsulate was weighed into a 15 mL centrifuge tube. The tubes were sealed and placed in a climate chamber for stability testing ICH260L (Memmert, Schwabach, Germany) at 60 °C. The samples were incubated for 72 h at a constantly controlled temperature without light. After 72 h, 10 mL of 0.1 M HCl was added to the tubes. The tubes were sealed and shaken in a laboratory rotator at room temperature at 80 rpm for 30 min. Samples before (initial samples) and after incubation were analyzed for total anthocyanins and phenolic compounds by spectrophotometric methods.

UV absorption spectra of the obtained solutions in the range of 400–700 nm were recorded to determine the stability index. The color stability index of the preparations was calculated based on the ratio of absorbance values at λ_max_ for the sample before and after the storage experiment from the equation [21]:

(1) $$Color\;stability\;index=\frac{Abs\lambda max\;after\;incubation}{Abs\lambda max\;initial}$$

### 3.9. Stability of Anthocyanins Under UV-A Irradiation

The stability of anthocyanins in the form of obtained extracts and encapsulates from cornflower flowers of the blue and pink varieties under UV-A irradiation was carried out using the method described by Bąkowska et al. (2003) [33], with some modification. For this purpose, about 0.0600 g of dried extract or encapsulate was weighed into a 15 mL centrifuge tube. A total of 15 mL of 0.1 M HCl was added to the tubes. The tubes were sealed and shaken in a laboratory rotator at room temperature at 80 rpm for 10 min. Then, a portion (5 mL) of each of the resulting 4 mg/mL solutions was transferred to 50 mL glass beakers. Then, samples were placed in a climate chamber for stability testing ICH260L equipped with a UV lamp in the spectral range of 320–400 nm (Memert, Germany) After turning on the UV-A lamp, the samples were incubated for 3 h. Samples before (initial samples) and after incubation were analyzed for total anthocyanins and phenolic compounds by spectrophotometric methods. The color stability index was also determined according to the procedure described in Section 3.8.

### 3.10. Statistical Analysis

The results of the three independent experiments were expressed as the mean ± standard deviation (SD). The Statistica 13.0 software (StatSoft, Inc., Tulsa, OK, USA) was used to analyze the statistical data. Significant differences were estimated using one-way analysis of variance (ANOVA) followed by the Tukey’s test (HSD). Differences were considered significant when *p*-values were less than 0.05 (*p* < 0.05).

## 4. Conclusions

The results showed that anthocyanins isolated from blue and pink cornflower flowers undergo copigmentation reactions with chlorogenic acid and baicalein. The greatest increase in absorbance intensity at wavelengths characteristic of the anthocyanins present in the extracts studied was found when baicalin was used as a copigment. The developed methods of encapsulation and copigmentation allow to increase the persistence of anthocyanins under the influence of high temperature and UV radiation. In comparison with the extracts from cornflower flowers, the color of the obtained encapsulates, regardless of the method and conditions of their preparation, also undergoes less changes. In addition, it has been shown that the use of baicalein as a copigment allows the greatest stabilization of the color of the obtained preparations. The use of the encapsulation and copigmentation process allows for a significant improvement in the anthocyanin color stability index after exposure to both heat and light. The anthocyanin encapsulates of pink cornflower flowers obtained by spray-drying with a copigment and a weight ratio of anthocyanin extract to carrier of 1:10 showed the highest stability. The highest loss of total anthocyanins and total phenolic compounds was observed in extracts and then in encapsulates where the weight ratio of extract to carrier was 1:4.

The obtained preparations were characterized by a high content of anthocyanins and phenolic compounds and strong antioxidant potential.

These results suggest that cornflower anthocyanin preparations, after encapsulation and copigmentation with baicalin or chlorogenic acid, may be used as stable colorants in the food industry, as well as functional food or cosmetic ingredients providing red–violet colors and high levels of antioxidants.

## Figures and Tables

**Table 1:** Identification of anthocyanins and main phenolic compounds in extracts from blue and pink cornflower flowers.

Peak No.	Rt (min)	[M+H]^+^ (*m/z*)	Fragment Ions (*m/z*)	Identified Compound	Abbreviation	EABC	EAPC
1	12.41	611.15	449.10, 287.05	Cyanidin 3,5-*O*-diglucoside	Cy3G5G	+	−
2	14.13	595.16	433.11, 271.06	Pelargonidin 3,5-di-O-glucoside	Pg3G5G	−	+
3	14.25	353.09 *	191.06	Chlorogenic acid	ChA	+	+
4	17.28	697.15	535.10, 449.10, 287.05	Cyanidin 3-*O*-(6″-malonyl-glucoside)-5-*O*-glucoside	Cy3malonylG5G	+	+
5	19.18	681.16	519.11, 433.11, 271.06	Pelargonidin 3-*O*-(6″-malonyl-glucoside)-5-*O*-glucoside	Pg3malonylG5G	−	+
6	19.56	711.17	549.12, 449.10, 287.05	Cyanidin 3-O-(6″-*O*-succinyl-glucoside)-5-*O*-glucoside	Cy3succinylG5G	+	−
7	21.77	695.17	533.12, 433.11, 271.06	Pelargonidin 3-O-(6″-*O*-succinyl-glucoside)-5-*O*-glucoside	Pg3succinylG5G	−	+
8	25.78	767.12	519.11, 433.11, 271.06	Pelargonidin 3-*O*-malonyl-glucoside-5-*O*-malonyl-glucoside	Pg3malonylG5malonylG	−	+
9	26.87	781.17	533.12, 519.11, 433.11, 271.07	Pelargonidin 3-*O*-malonyl-glucoside-5-*O*-succinyl-glucoside	Pg3malonylG5succinylG	−	+
10	31.79	447.08 *	271.06	Apigenin 7-*O*-glucuronide	Ap-GluA	+	+

Rt—retention time; * [M−H]^−^—molecular ion detected in negative mode (*m*/*z*); EABC—extract from blue cornflower flowers; EAPC—extract from pink cornflower flowers.

**Table 2:** Total anthocyanin content, total phenolic content, and antioxidant activity of extracts from blue and pink cornflower flowers.

	EABC	EAPC
Total anthocyanins (mg Cy3G/g dw)	15.78 ± 0.76 a	60.00 ± 1.60 b
Total phenolic compounds (mg GA/g dw)	96.32 ± 0.55 a	411.17 ± 2.35 b
TEAC_DPPH_ (μM TEg dw)	402.19 ± 2.30 a	511.88 ± 2.93 b
TEAC_FRAP_ (μM TE/g dw)	383.47 ± 1.78 a	485.81± 1.56 b

Data are presented as mean ± SD of three replicates. Different lowercase letters in the same row indicate statistically significant differences according to Tukey’s HSD test at *p* < 0.05. EABC—extract from blue cornflower flowers. EAPC—extract from pink cornflower flowers. Total anthocyanins are expressed as milligrams of cyanidin 3-*O*-glucoside per gram of dry weight (mg Cy3G/g dw). Total phenolic content was determined using the Folin–Ciocalteu assay and expressed as milligrams of gallic acid per gram of dry weight (mg GA/g dw). The antioxidant capacity (TEAC_DPPH_ and TEAC_FRAP_) was expressed as µM Trolox equivalents per gram of dry weight (μM TE/g dw).

**Table 3:** Total anthocyanin content, total phenolic content, and antioxidant activity of blue and pink cornflower anthocyanin encapsulates.

Sample	TAC (mg Cy3G/g dw)	TPC (mg GA/g dw)	TEAC_DPPH_ (μM TE/g dw)	TEAC_FRAP_ (μM TE/g dw)
EN-ABC-MAF-1:4-SD	3.93 ± 0.05 b	80.49 ± 0.15 c	319.53 ± 1.05 e	307.70 ± 1.12 g
EN-ABC-MAF-1:4-FD	4.03 ± 0.04 b	94.46 ± 0.14 d	375.01 ± 1.13 f	361.13 ± 1.78 h
EN-ABC-B-MAF-1:4-SD	3.93 ± 0.07 b	112.68 ± 0.16 f	447.34 ± 0.95 i	430.79 ± 1.45 j
EN-ABC-ChA-MAF-1:4-SD	4.03 ± 0.06 b	109.45 ± 0.13 e	434.53 ± 0.89 h	418.45 ± 1.43 i
EN-ABC-B-MAF-1:4-FD	3.91 ± 0.05 b	125.07 ± 0.12 h	496.53 ± 0.78 k	478.16 ± 1.27 l
EN-ABC-ChA-MAF-1:4-FD	4.01 ± 0.07 b	123.53 ± 0.11 g	490.42 ± 0.65 j	472.27 ± 1.39 k
EN-ABC-MAF-1:10-SD	1.57 ± 0.05 a	51.11 ± 0.08 a	128.71 ± 1.02 a	138.32 ± 1.38 a
EN-ABC-MAF-1:10-FD	1.61 ± 0.03 a	51.77 ± 0.11 a	130.92 ± 0.85 a	140.53 ± 1.51 b
EN-ABC-B-MAF-1:10-SD	1.57 ± 0.02 a	77.32 ± 0.12 b	187.53 ± 0.92 c	203.73 ± 1.34 d
EN-ABC-ChA-MAF-1:10-SD	1.61 ± 0.05 a	76.37 ± 0.11 b	176.34 ± 0.95 b	194.40 ± 1.41 c
EN-ABC-B-MAF-1:10-FD	1.57 ± 0.04 a	79.94 ± 0.12 c	195.79 ± 0.91 d	212.10 ± 1.30 f
EN-ABC-ChA-MAF-1:10-FD	1.61 ± 0.03 a	77.98 ± 0.15 b	189.67± 0.78 c	205.88 ± 1.29 e
EN-APC-MAF-1:4-SD	14.93 ± 0.09 c	171.79 ± 0.16 f	428.95 ± 0.84 g	316.18 ± 1.27 f
EN-APC-MAF-1:4-FD	15.31 ± 0.05 d	201.62 ± 0.17 g	503.44 ± 0.78 h	371.08 ± 1.23 g
EN-APC-B-MAF-1:4-SD	14.93 ± 0.04 c	240.50 ± 0.19 i	600.54 ± 0.65 j	442.65 ± 1.42 i
EN-APC-ChA-MAF-1:4-SD	15.31 ± 0.08 d	232.35 ± 0.18 h	580.18 ± 0.76 i	427.65 ± 1.28 h
EN-APC-B-MAF-1:4-FD	14.87 ± 0.07 c	266.95 ± 0.21 k	666.58 ± 0.98 l	491.33 ± 1.54 k
EN-APC-ChA-MAF-1:4-FD	15.25 ± 0.05 d	257.90 ± 0.17 j	643.98 ± 1.05 k	474.68 ± 1.34 j
EN-APC-MAF-1:10-SD	5.97 ± 0.02 a	89.77 ± 0.15 a	122.66 ± 0.67 a	111.81 ± 1.16 a
EN-APC-MAF-1:10-FD	6.12 ± 0.03 a,b	93.37 ± 0.11 b	128.56 ± 0.98 b	116.81 ± 1.33 b
EN-APC-B-MAF-1:10-SD	5.97 ± 0.07 a	112.05 ± 0.13 d	206.40 ± 0.67 d	167.61 ± 1.62 c
EN-APC-ChA-MAF-1:10-SD	6.12 ± 0.05 a,b	113.76 ± 0.12 e	204.56 ± 0.58 c	167.54 ± 1.45 c
EN-APC-B-MAF-1:10-FD	5.95 ± 0.06 a	108.45 ± 0.15 c	227.63 ± 0.85 f	176.88 ± 1.12 e
EN-APC-ChA-MAF-1:10-FD	6.10 ± 0.03 a,b	107.63 ± 0.13 c	220.98 ± 0.76 e	172.95 ± 1.23 d

Data are presented as mean ± SD of three replicates. Different lower-case letters (a–l) in the same column for different varieties of cornflower indicate differences that are statistically significant according to Tukey’s HSD test at *p* < 0.05. TAC—total anthocyanins are expressed as mg Cy3G/g dw. TPC—total phenolic content expressed mg GA/g dw. TEAC_DPPH_—total antioxidant capacity expressed μM TE/g dw. TEAC_FRAP_—total antioxidant capacity expressed μM TE/g dw.

**Table 4:** Index of color stability of cornflower anthocyanin extracts and encapsulates during thermal and light treatments.

Sample	Color Stability Index	
	Thermal Treatment	Light Treatment
EABC	0.61 ± 0.03 a	0.72 ± 0.02 a
EN-ABC-MAF-1:4-SD	0.84 ± 0.01 a	0.85 ± 0.01 b
EN-ABC-MAF-1:4-FD	0.83 ± 0.01 a	0.84 ± 0.02 b
EN-ABC-B-MAF-1:4-SD	0.86 ± 0.02 b	0.87 ± 0.02 b,c
EN-ABC-ChA-MAF-1:4-SD	0.86 ± 0.02 b	0.87 ± 0.01 b,c
EN-ABC-B-MAF-1:4-FD	0.83 ± 0.03 a	0.85 ± 0.01 b
EN-ABC-ChA-MAF-1:4-FD	0.82 ± 0.02 a	0.84 ± 0.02 b
EN-ABC-MAF-1:10-SD	0.86 ± 0.02 b	0.87 ± 0.02 b,c
EN-ABC-MAF-1:10-FD	0.85 ± 0.01 b	0.86 ± 0.02 b
EN-ABC-B-MAF-1:10-SD	0.91 ± 0.01 d	0.93 ± 0.01 d
EN-ABC-ChA-MAF-1:10-SD	0.90 ± 0.02 d	0.92 ± 0.01 d
EN-ABC-B-MAF-1:10-FD	0.87 ± 0.02 b,c	0.85 ± 0.02 b
EN-ABC-ChA-MAF-1:10-FD	0.87 ± 0.02 b,c	0.84 ± 0.02 b
EAPC	0.70 ± 0.01 a	0.86 ± 0.01 a
EN-APC-MAF-1:4-SD	0.85 ± 0.01 b	0.87 ± 0.03 a
EN-APC-MAF-1:4-FD	0.84 ± 0.03 b	0.86 ± 0.01 a
EN-APC-B-MAF-1:4-SD	0.87 ± 0.02 c	0.89 ± 0.01 b
EN-APC-ChA-MAF-1:4-SD	0.87 ± 0.02 c	0.89 ± 0.02 b
EN-APC-B-MAF-1:4-FD	0.85 ± 0.01 b	0.86 ± 0.01 a
EN-APC-ChA-MAF-1:4-FD	0.84 ± 0.01 b	0.86 ± 0.02 a
EN-APC-MAF-1:10-SD	0.90 ± 0.01 d	0.91 ± 0.02 b,c
EN-APC-MAF-1:10-FD	0.87 ± 0.02 c	0.89 ± 0.02 b
EN-APC-B-MAF-1:10-SD	0.96 ± 0.02 f	0.97 ± 0.03 e
EN-APC-ChA-MAF-1:10-SD	0.94 ± 0.02 e	0.94 ± 0.02 d
EN-APC-B-MAF-1:10-FD	0.89 ± 0.01 c,d	0.92 ± 0.02 c
EN-APC-ChA-MAF-1:10-FD	0.87 ± 0.02 c	0.91 ± 0.02 b,c

Data are presented as mean ± SD of three replicates. Different lower-case letters (a–f) in the same column for different varieties of cornflower indicate differences that are statistically significant according to Tukey’s HSD test at *p* < 0.05.

## Data Availability

Data is contained within the article.

## Abbreviations

The following abbreviations are used in this manuscript:

EABC	Extract of anthocyanins from the blue cornflower flowers
EAPC	Extract of anthocyanins from the pink cornflower flowers
EN-ABC-MAF-1:4-SD	Encapsulate of EABC with maltodextrin and acacia fiber (1:1) in a weight ratio of 1:4 obtained by spray-drying
EN-ABC-MAF-1:4-FD	Encapsulate of EABC with maltodextrin and acacia fiber (1:1) in a weight ratio of 1:4 obtained by freeze-drying
EN-ABC-B-MAF-1:4-SD	Encapsulate of EABC and baicalin with maltodextrin and acacia fiber (1:1) in a weight ratio of 1:4 obtained by spray-drying
EN-ABC-ChA-MAF-1:4-SD	Encapsulate of EABC and chlorogenic acid with maltodextrin and acacia fiber (1:1) in a weight ratio of 1:4 obtained by spray-drying
EN-ABC-B-MAF-1:4-FD	Encapsulate of EABC and baicalin with maltodextrin and acacia fiber (1:1) in a weight ratio of 1:4 obtained by freeze-drying
EN-ABC-ChA-MAF-1:4-FD	Encapsulate of EABC and chlorogenic acid with maltodextrin and acacia fiber (1:1) in a weight ratio of 1:4 obtained by freeze-drying
EN-ABC-MAF-1:10-SD	Encapsulate of EABC with maltodextrin and acacia fiber (1:1) in a weight ratio of 1:10 obtained by spray-drying
EN-ABC-MAF-1:10-FD	Encapsulate of EABC with maltodextrin and acacia fiber (1:1) in a weight ratio of 1:10 obtained by freeze-drying
EN-ABC-B-MAF-1:10-SD	Encapsulate of EABC and baicalin with maltodextrin and acacia fiber (1:1) in a weight ratio of 1:10 obtained by spray-drying
EN-ABC-ChA-MAF-1:10-SD	Encapsulate of EABC and chlorogenic acid with maltodextrin and acacia fiber (1:1) in a weight ratio of 1:10 obtained by spray-drying
EN-ABC-B-MAF-1:10-FD	Encapsulate of EABC and baicalin with maltodextrin and acacia fiber (1:1) in a weight ratio of 1:10 obtained by freeze-drying
EN-ABC-ChA-MAF-1:10-FD	Encapsulate of EAPC and chlorogenic acid with maltodextrin and acacia fiber (1:1) in a weight ratio of 1:10 obtained by freeze-drying
EN-APC-MAF-1:4-SD	Encapsulate of EAPC with maltodextrin and acacia fiber (1:1) in a weight ratio of 1:4 obtained by spray-drying
EN-APC-MAF-1:4-FD	Encapsulate of EAPC with maltodextrin and acacia fiber (1:1) in a weight ratio of 1:4 obtained by freeze-drying
EN-APC-B-MAF-1:4-SD	Encapsulate of EAPC and baicalin with maltodextrin and acacia fiber (1:1) in a weight ratio of 1:4 obtained by spray-drying
EN-APC-ChA-MAF-1:4-SD	Encapsulate of EAPC and chlorogenic acid with maltodextrin and acacia fiber (1:1) in a weight ratio of 1:4 obtained by spray-drying
EN-APC-B-MAF-1:4-FD	Encapsulate of EAPC and baicalin with maltodextrin and acacia fiber (1:1) in a weight ratio of 1:4 obtained by freeze-drying
EN-APC-ChA-MAF-1:4-FD	Encapsulate of EAPC and chlorogenic acid with maltodextrin and acacia fiber (1:1) in a weight ratio of 1:4 obtained by freeze-drying
EN-APC-MAF-1:10-SD	Encapsulate of EAPC with maltodextrin and acacia fiber (1:1) in a weight ratio of 1:10 obtained by spray-drying
EN-APC-MAF-1:10-FD	Encapsulate of EAPC with maltodextrin and acacia fiber (1:1) in a weight ratio of 1:10 obtained by freeze-drying
EN-APC-B-MAF-1:10-SD	Encapsulate of EAPC and baicalin with maltodextrin and acacia fiber (1:1) in a weight ratio of 1:10 obtained by spray-drying
EN-APC-ChA-MAF-1:10-SD	Encapsulate of EAPC and chlorogenic acid with maltodextrin and acacia fiber (1:1) in a weight ratio of 1:10 obtained by spray-drying
EN-APC-B-MAF-1:10-FD	Encapsulate of EAPC and baicalin with maltodextrin and acacia fiber (1:1) in a weight ratio of 1:10 obtained by freeze-drying
EN-APC-ChA-MAF-1:10-FD	Encapsulate of EAPC and chlorogenic acid with maltodextrin and acacia fiber (1:1) in a weight ratio of 1:10 obtained by freeze-drying
